# Anatomical and Physiological Remarks on the Connexion between the Placenta and Uterus: On the Vascular Communications between These Organs, and the Mode in Which Their Fluids Circulate

**Published:** 1833-05

**Authors:** M. E. A. Lauth


### CONNEXION BETWEEN THE PLACENTA AND UTERUS.

*Anatomical and Physiological Remarks on the Connexion between the Placenta and Uterus: on the Vascular Communications between these Organs, and the Mode in which their Fluids circulate.* By M. E. A. Lauth, fils, M.D.—*Repert. d'Anatomie et de Physiologie*, t. i. p. 226.‡

The mode of union between the uterus and the placenta, and the reciprocal exchange of blood between the mother and the fœtus, have long excited the attention of anatomists and physiologists. Neither, however, have succeeded in giving us clear ideas of the structure of these parts, nor of the manner in which the functions that depend upon them are performed. When the discovery of the circulation of the

‡ We give the translation of M. Lauth's paper, for the purpose of affording our readers an opportunity of comparing it with that of Dr. R. Lee, which we published in our number for July 1832, p. 71. Dr. Granville lately declared, at the Westminster Medical Society, that M. Lauth's paper was an "anticipated translation" of Dr. Lee's. We maintained then, and we still remain of the same opinion, notwithstanding Dr. Granville's "Parallel," (*Lancet*, March 23,) that there is no more similarity between these papers than must necessarily exist when two persons describe the appearances of the same thing which they have both carefully examined. It cannot escape observation, that the object of the two writers is essentially different.—Editors.

blood was made, an intermediate parenchyma was admitted to exist between the uterus and the placenta, at the extremities of the arteries and the commencement of the veins, as in all other parts of the body. But microscopic observations and injections having demonstrated the non-existence of a parenchyma in the rest of the body, it was inferred, from analogy, that there was an immediate continuation of the uterine arteries into the veins of the placenta, and of the arteries of the placenta into the veins of the uterus. It was soon found, however, that it was impossible to inject the vessels of the fœtus through those of the mother, and *vice versâ*. The injection thrown into the arteries was always arrested between the placenta and uterus, and formed extravasations, more or less considerable, in the interstice between these two organs, when it had been thrown in with too much force; or it returned by the veins if the injection was sufficiently thin, and the operation carefully conducted. The frequent repetition of these experiments, and always with the same results, only rescued physiologists from one error, to plunge them into another. An immediate connexion between the vessels of the uterus and placenta was no longer admitted; it was now asserted that the arteries terminated by open orifices in the cells situated between the two organs, or in the uterine portion of the placenta, and that the veins there absorbed the effused blood; those of the placenta, to conduct it to the fœtus, and those of the uterus, to carry it back to the mother.

In the experiments I have made upon this subject, my first object was to examine what was to be understood by fœtal placenta and uterine placenta; portions which, according to modern anatomists, are composed of totally different vessels; the one being the prolongations of the uterine vessels, and the others nothing more than the expansion of the vessels which compose the umbilical cord. Notwithstanding the most minute examinations, I could not discover these two portions, unless the term uterine placenta is applied to that portion of the membrana decidua to which the placenta has become adherent, and which, after its more or less complete separation from the uterus, is expelled with the placenta, the external surface of which it covers. The formation of the membrana decidua in the uterus before the descent of the ovum into that viscus, and even independently of its descent in cases of extra-uterine pregnancy, proves that it is a production of the womb, and that, though the placenta subsequently forms adhesions with the decidua, it ought not to be considered as forming a body with it, or as constituting its uterine portion.

There does not, then, exist a uterine placenta, such as it has hitherto been described. The membrana decidua, like most false membranes, when it becomes organized, receives numerous vessels, which are the continuations of the uterine vessels, or which are, at least, in direct communication with them; and, as in all other parts of the body, the final extremities of the arteries are there reflected upon themselves to form the commencement of the veins. The placenta is entirely composed of the successive division of the umbilical arteries upon the chorion, which arteries, having arrived at the extremities of the villosities which cover the chorion, are reflected upon themselves to give origin to the veins, and the trunk which results from their reunion is the umbilical vein. I have frequently seen, without the assistance of the microscope, this termination of the arteries in the veins, in human and animal placentas, of which the blood-vessels had been previously injected. The artery turns abruptly upon itself, to be changed into a vein, in such a manner as to form a very small arch. The termination of the arteries in the veins of the placenta may easily be seen by the injection of a thin liquid, as coloured water, which is readily made to pass from one order of vessels into the other. These blood-vessels of the placenta have no direct communication with those of the membrana decidua. No one has ever succeeded in injecting the one through the other, however subtle might be the nature of the injecting liquid that was employed. It is very easy to repeat this experiment upon the placenta, provided it is not torn nor injured: not an atom of the injected matter will penetrate into the vessels of the decidua which covers it, and there will be no injection extravasated, unless sufficient violence has been used to tear the parts.

This experiment proves, at the same time, that the passage of the blood of the mother to the fœtus cannot be effected by means of what have been called the absorbing radicles of veins, as these would necessarily be torn in a placenta detached from the uterus, and the injected matter would be extravasated, which we have just seen is not the case; but that the transmission of the blood is effected by means of particular vessels, in the interior of which there are valves which only permit the blood to enter under certain circumstances, and under the influence of vital laws.

In carefully examining a placenta still covered by the decidua, we see that these two parts are united together by a multitude of small transparent vessels, which run from the one to the other. These vessels cannot be injected through the placental vessels, nor through those of the decidua; but a fine tube, introduced into either, enables us to fill sometimes the vessels of the placenta, and sometimes those of the decidua. Hence it follows, 1st, that there are two orders of these vessels, the one belonging to the membrana decidua, and consequently to the uterus, and the other to the placenta; 2d, that they are not blood-vessels; and, 3d, that they terminate, the one order in the blood-vessels of the decidua, and the other in those of the placenta, by orifices furnished with valves, which prevent their being injected in a retrograde way. The vessels of which I speak can only be lymphatic branches, of which they present all the characters: they do not, however, appear to be connected with the general lymphatic system, because they are grafted upon temporary organs, with which they are expelled when the placenta is removed from the uterus.

As to the cells which are described between the uterus and placenta, or in the uterine portion of the latter, where the arteries terminate, and where the veins arise to absorb the blood there effused, I have never been able to discover them, notwithstanding the attention with which I have made my examinations. The circumstance which may have given rise to the belief of the existence of these cells is the occasional formation of extravasations of the injected matter between the two organs; but these extravasations arise from the separation of the placenta and the membrana decidua, and the rupture of the vessels which unite it to the uterus; and they are sometimes so extensive as to leave the placenta adhering to the uterus by its edges alone. And it is also probable that blood-vessels, very much dilated, as is the case in all organs the vital properties of which are preternaturally excited, have been taken for cells.

As it is now proved that there is no direct communication between the uterine and placental vessels, and as we have just stated that the cells in which the blood would be effused do not exist, the only communication we can admit between the mother and the fœtus is that by lymphatic vessels, which have been described above, and some of which terminate in the vessels of the placenta, and others in those of the membrana decidua, which are themselves nothing more than prolongations of the uterine vessels. These lymphatics, which terminate in the blood-vessels of one of these organs, appear to be engrafted by their origins on those of the other; and thus those which arise from the uterine vessels, and which terminate in the vessels of the placenta, extract from the blood of the mother the materials which are fitted to enter into the composition of the blood of the fœtus. The fœtal blood is elaborated and accommodated to the wants of the fœtus as it passes through the liver, where it appears to receive an arterial character. On the other hand, the lymphatic radicles grafted upon the vessels of the placenta terminate in the uterine vessels, and serve to remove from the blood of the fœtus the materials which are no longer useful, or which might become injurious to it, and to convey them into the venous system of the mother.

Certain arguments also tend to support this opinion. The difference that exists between the movements of the blood in the mother and in the fœtus, appears to prove that their sanguineous systems are connected together by a totally different order of vessels. The same conclusion must be drawn from the reciprocal independence which exists in the health of the mother and that of the fœtus. Experience teaches us that an unhealthy mother may bring into the world a healthy and robust child; that the mother may be affected with syphilis, or smallpox, without the child being affected; and, on the other hand, that the child may be born with various diseases from which the mother is perfectly free. I think it must be admitted, without incurring the reproach of humoral doctrines, that in all these cases the blood which has passed from one individual to another, must have undergone essential modifications, and must have been changed by absorbent vessels, by some mechanism analogous to that by virtue of which a portion of the chymous mass, for example, is transformed into chyle by passing through the lacteals. The analogy furnished by the examination of the incubated egg, forms an additional proof that the new being does not derive its development from the successive addition of portions of blood already formed, but that the blood is elaborated by the embryo itself, or by its dependencies, and that it secretes from the surrounding nutritive substance the necessary materials for the composition of the blood.*

The last argument I shall adduce in support of this opinion is the experiment of the transfusion of blood, which shews that the blood of one individual cannot be altogether suitable for another, as is proved by the serious symptoms which have followed this operation. The losses which the body sustains cannot be repaired by the simple addition of a certain quantity of blood: it is necessary that the mass of the blood should be augmented by a fluid elaborated by lymphatic vessels; vessels which are found in the placenta before

* Some recent microscopical experiments have shewn that the globules of blood of the mother do not resemble those of the fœtus. G. B., *Repert. d'Anat.*

birth, and which after birth are replaced by those of the intestines, the lacteals.

We see then, *en resumé*, that the union of the placenta with the uterus is formed by vessels which are not blood-vessels, and which present all the characters of lymphatics; that the function in question is a positive act of absorption; that this absorption cannot take place by means of veins, because venous absorption, if it exists at all, only takes place by transudation; and that, consequently, this function must be performed by the vessels I have described, because no other than vessels of the lymphatic kind are capable of modifying the blood of the mother so as to accommodate it to the wants of the fœtus; and, lastly, that the placenta appears to fulfil in the fœtus the functions which are subsequently performed by the intestinal canal, rather than the functions of lungs, which have been hitherto attributed to it.

